# Calcium Treatment Alleviates Pericarp Browning of ‘Nanguo’ Pears by Regulating the GABA Shunt After Cold Storage

**DOI:** 10.3389/fpls.2020.580986

**Published:** 2020-09-15

**Authors:** Jiaxin Li, Qian Zhou, Xin Zhou, Baodong Wei, Yingbo Zhao, Shujuan Ji

**Affiliations:** Research Laboratory of Postharvest Biology and Storage and Preservation for Fruits and Vegetables, Department of Food Science, Shenyang Agricultural University, Shenyang, China

**Keywords:** ‘Nanguo’ pear, GABA, Ca^2+^, pericarp browning, chilling injury, cold storage

## Abstract

Long-term storage of pear fruit at low temperature can retard senescence but may result in pericarp browning. We previously reported that increasing endogenous γ-aminobutyrate (GABA) content by exogenous GABA treatment can maintain mitochondrial structure integrity, thereby alleviating pericarp browning of ‘Nanguo’ pears after cold storage. Here, we tested the effectiveness of Ca^2+^ treatment on pericarp browning in relation to GABA biosynthesis. Fruit browning was reduced by treatment with Ca^2+^ after 180 days of storage. Pericarp Ca^2+^ and calmodulin content in treated fruit increased, and concomitantly, endogenous GABA content, key GABA synthesis-related enzyme activity, and gene expression were upregulated. Moreover, the mitochondrial structure in the pericarp tissue was found to be well preserved. Thus, Ca^2+^ treatment effectively reduced pericarp browning of refrigerated ‘Nanguo’ pears owing to improvement in the GABA biosynthesis capacity in the fruit.

## Introduction

‘Nanguo’ pear (*Pyrus ussuriensis* Maxim.) is one of the most famous pear varieties in Liaoning Province, China, where it is harvested in September each year. Unfortunately, the fruit ripen and senesce rapidly at room temperature, resulting in pulp softening and browning of the core. Therefore, ‘Nanguo’ pear fruit are commonly refrigerated while being stored. However, long-term cold storage renders the fruit prone to peel browning during shelf life at room temperature, even if the fruit has not ripened or softened (Wang et al., 2017).

Fruit browning after refrigeration signals metabolic disorders owing to low temperature stress, such as an energy deficit because of the cold-induced reduction of ATP production. Reduced ATP production is associated with damage of the mitochondrial structures and inhibition of enzymes involved in mitochondrial function (Atkin and Tjoelker, 2003). Mitochondria are the source of all metabolic energy in plant cells. Maintaining the integrity of mitochondrial structure plays a crucial role in controlling peel browning in postharvest ‘Nanguo’ pear. Wang et al. (2018) found that an intermittent warming during cold storage reduces pericarp browning of ‘Nanguo’ pears after a 120-day storage period, which is associated with modified energy and lipid metabolism. Furthermore, exogenous melatonin treatment retards the increase in cell membrane permeability and maintains a high energy state, thereby delaying the browning of litchi fruit after harvest (Wang et al., 2020). Similarly, Cheng et al. (2015) found that treatment with 1-MCP increases the activity of enzymes related to energy metabolism and alleviates the occurrence of chilling injury in ‘Nanguo’ pears.

The non-protein amino acid, γ-Aminobutyrate (GABA), helps plant cells resist biotic and abiotic stress (Fait et al., 2008). GABA content in plant tissues is relatively low; however, it rapidly increases under abiotic stress, such as chilling, salinity, and drought (Mekonnen et al., 2016; Salvatierra et al., 2016; Li J. X. et al., 2019). Morteza et al. (2016) found that GABA treatment reduces chilling injury in *Anthurium* cut flowers by increasing the activity of the GABA shunt in order to provide sufficient energy. Reduced cold injury in bananas by GABA is associated with the accumulation of proline and triggers the antioxidant defense system (Wang et al., 2014). GABA immersing can significantly inhibit the browning process of fresh-cut apples during postharvest storage (Gao et al., 2018).

Li J. X. et al. (2019) observed an increase in the endogenous GABA content in ‘Nanguo’ pears treated with GABA. Moreover, GABA treatment enhances the mitochondrial antioxidant response in treated pears, whereby the mitochondrial structure remains intact, and, pericarp browning of the fruit after a 180-day storage is effectively retarded (Li J. X. et al., 2019). GABA is synthesized primarily in plant tissues by the cytoplasmic α-decarboxylation of glutamic acid in an irreversible reaction of the GABA shunt, catalyzed by GABA decarboxylase (GAD), whose activity is regulated by Ca^2+^ and calmodulin (CML). GABA is then transported into the mitochondria, where it is converted to succinic semialdehyde (SSA) by GABA transaminase (GABA-T). In the final step of the synthesis, SSA is reduced by succinate semialdehyde dehydrogenase (SSADH) to form succinic acid, which enters the tricarboxylic acid (TCA) cycle (Guo et al., 2012). Yin et al. (2014) found that CaCl_2_ can reduce the harmful effects of soybeans under NaCl stress by changing the contribution ratio of GABA shunt and promoting GABA production.

Ionic calcium (Ca^2+^) is abundantly available in plants and acts as a secondary messenger during plant growth and development. Ca^2+^ plays an important regulatory role in vital processes, such as cell division, polar cell growth, cell differentiation, and apoptosis (Poovaiah and Ready, 1993). Exogenous Ca^2+^ application provides plants the ability to tolerate chilling related stress. Postharvest Ca^2+^ application reduces the severity of chilling injury in fruit flesh by increasing flesh calcium, which in turn delays fruit browning after cold storage (Manganaris et al., 2007). GAD has been identified as a CAM-binding protein in plants, stimulated by Ca^2+^/CAM (Baum et al., 1993; Ling et al., 1994; Kinnersley and Turano, 2000). Ca^2+^-based signals in plants involve a complex mix of different binding proteins that function as Ca^2+^ sensors, such as CAM, CML, Ca^2+^-dependent protein kinase (CDPK), CML B-like protein (CBL), and CBL-interaction kinase (CIPK). These Ca^2+^ sensors constitute a complex signal network in plants enabling specific signal transduction (Batistič and Kudla, 2012). Many previous studies have shown that calcium can promote the accumulation of GABA (Gao et al., 2011; Bai et al., 2013).

In view of the function of GABA in maintaining the integrity of the mitochondrial structure and energy production capacity, and given the relationship between GABA biosynthesis and Ca^2+^, further insight into the protective mechanism mediated by GABA and Ca^2+^ against cold injury in pear fruit will require a thorough study of Ca^2+^, CAM, CML, and GABA contents and distribution in pericarp tissues, as well as GABA shunt-related enzyme activities and gene expression. Here, we studied the effects of exogenous Ca^2+^ treatment on pericarp browning in ‘Nanguo’ pears.

## Material and Methods

### Fruit Material and Postharvest Treatments

‘Nanguo’ pears were collected from commercial orchards in Anshan, Liaoning Province, China on September 11, 2018. Sampled fruit were transported to the laboratory within 3 h of harvest. Uniformly sized and matured fruit without decay or damage were selected and packed in 0.04 mm thick polyethylene bags. The pears were placed at room temperature (20 ± 1°C) for 4 days for pre-ripening and then divided randomly into two groups, each consisting of 600 fruit. The fruit in treated group were soaked in CaCl_2_ solution (4%) for 10 min (**Supplementary Figure 1**), and the fruit in the control group were soaked in distilled water for 10 min. Three independent biological replicates were set. After naturally air-drying, all fruit were precooled for 24 h at 0°C and packed in 0.04-mm-thick polyethylene (PE) bags for storage at 0 ± 0.5°C and 80% to 90% relative humidity (RH). The pears were removed from cold storage and shelved at 20 ± 1°C and 80% to 85% RH after 180 days of cold storage. Triplicate samples were drawn from the experimental and control groups at 3-day intervals after cold storage.

### Evaluation of Browning Index

The browning index (BI) of the pericarp was evaluated according to the method described by Sheng et al. (2016). The extent of browning of the pericarp was classified into four grades according to the percentage area affected, as follows: 0 (no browning), Grade 1 (browning area ≤ 1/3), Grade 2 (1/3 < browning area ≤ 2/3), and Grade 3 (browning area > 2/3). BI was calculated using the following formula:

Equation 1 $$Browning\;index(\%)=\frac{\Sigma (grade\;number\;\times \;number\;of\;fruit\;corresponding\;to\;the\;grade)}{highest\;browning\;grade\;\times total\;number\;of\;fruit}\times 100,$$

### Determination of Firmness

The firmness of pears was measured using TA.XT*plusC* Texture Analyser (Stable Micro System, Guildford, UK) and the measuring conditions of the texture analyzer are: probe model P/2, test rate 3 mm/s; adjustment measuring arm 5 mm; trigger point load 0.2 N. Six fruit were randomly selected, and four points were taken at equal distances from the equator of the fruit to determine the firmness of the pears.

### Determination of Ca^2+^ Content and Calcium Distribution

Ca^2+^ content (mg·g^−1^) was measured using a calcium colorimetric assay kit (Beyotime Biotechnology Co., Ltd. Shanghai, China). Free Ca^2+^ was localized by fluorescence imaging, as previously described by Qu et al. (2012) with some modifications. Fruits were cut manually using a razor blade to a thickness of approximately 1 mm; 50 μg of Fluo-3-AM (Beyotime Biotechnology Co., Ltd. Shanghai, China) was dissolved in 45 μl (15 μl/min) of 1 mM dimethyl sulfoxide (DMSO) and stored at −20°C. Then, 1 mM Fluo-3-AM was diluted to 10 μM with HEPES-containing buffer, and 5 μl of Pluoronic F127 was added per mL of the stain. Slices were observed immediately after preparation. Ca^2+^ fluorescence signal image-acquisition was performed using a TSC SP8 laser scanning confocal microscope (Leica, Germany). The XYZ scan mode was selected using Argon excitation light (wavelength 488 nm) and transmitted light (DIC) scanning. Image files were saved at a resolution of 1024 × 1024 pixels.

### Determination of CAM and CML Content

Peel samples (2 g) were homogenized in 2 mL phosphate buffered saline (pH 7.4) at 4°C. After centrifugation at 13,000*g* for 15 min at 4°C, the supernatant was used for CAM and CML concentration assays. The concentration of CAM and CML (U/L) was calculated using a standard curve, as defined in the Plant CAM ELISA Kit and CML ELISA Kit (ZK Biotechnology Co. Ltd., Shenzhen, China) instructions, respectively.

### Determination of GABA Content

Peel samples (2 g) were homogenized in 2 mL phosphate buffered saline (pH 7.4) at 4°C. After centrifugation at 13,000*g* for 20 min at 4°C, the supernatant was used for GABA (g kg^−1^) assays. Measurements were performed according to instructions of the Plant γ-aminobutyrate (GABA) ELISA Kit (ZK, Shenzhen Biotechnology Co., Ltd. Shenzhen, China). Sample concentration was calculated using a standard curve, as defined in the kit instructions.

### Analysis of GAD, GABA-T, and SSADH Activities

GAD, GABA-T, and SSADH activities (U/L) were determined using plant enzyme activity kits (Shanghai Enzyme-linked Biotechnology Co., Ltd. Shanghai, China). Sample concentrations were calculated using a standard curve (according to the manufacturer’s instructions).

### RNA Isolation and cDNA Synthesis

Total RNA from frozen pears was extracted using an OmniPlant RNA extraction kit (CWBIO, Beijing, China) according to the manufacturer’s instructions. Total RNA purity and concentration purity were quantified at optical densities (OD; nm) of OD_260_/OD_230_ and OD_260_/OD_280_ using a microplate reader (Eon™, BioTek, USA). Total RNA integrity was determined using 1.0% agar-gel electrophoresis. Before reverse transcription, the total RNA was stored at −80°С. Subsequently, the HiFiScript cDNA Synthesis Kit (CWBIO, Beijing, China) was used to synthesize the first-strand cDNA, which, in turn, was used as a template for the quantitative reverse-transcription polymerase chain reaction (RT-qPCR) analysis.

### RT-qPCR Assay

RT-qPCR and the QuantStudio 6 Flex instrument (Life Technologies, Camarillo, CA, USA) were used to assess differences in gene expression. *PuActin* was used as an internal reference gene to correct for differences. Gene expression analysis was performed using RT-qPCR with RealMasterMix (CWBIO) on a QuantStudio 6 Flex system. The specific primers used in these experiments were designed using Primer 6.0 software (**Supplementary Table 1**). Three replicates of each sample were analyzed. The 2^-ΔΔCt^ calculation method for RT-qPCR was used.

### Ultrastructural Observation

For ultrastructural observation of mitochondria isolated from the pericarp, CaCl_2_-treated and untreated ‘Nanguo’ pears were selected on the 12^th^ day of shelf storage (after 180 days of cold-storage). Nine pears were randomly sampled in each group, and three pieces of pericarp (1 mm × 1 mm × 2 mm) were cut from the equator of each pear using a new scalpel blade. The prepared samples were observed using transmission electron microscopy (TEM), according to the method described by Li J. X. et al. (2019).

### Statistical Analysis

Statistical analyses were performed using SPSS software 16.0 (SPSS Inc.). Analysis of variance was conducted to check the significance of differences among samples. The mean interval was calculated using the least significant difference test. Differences were considered significant at P < 0.05.

## Results

### Pericarp Browning

**Figure 1:**
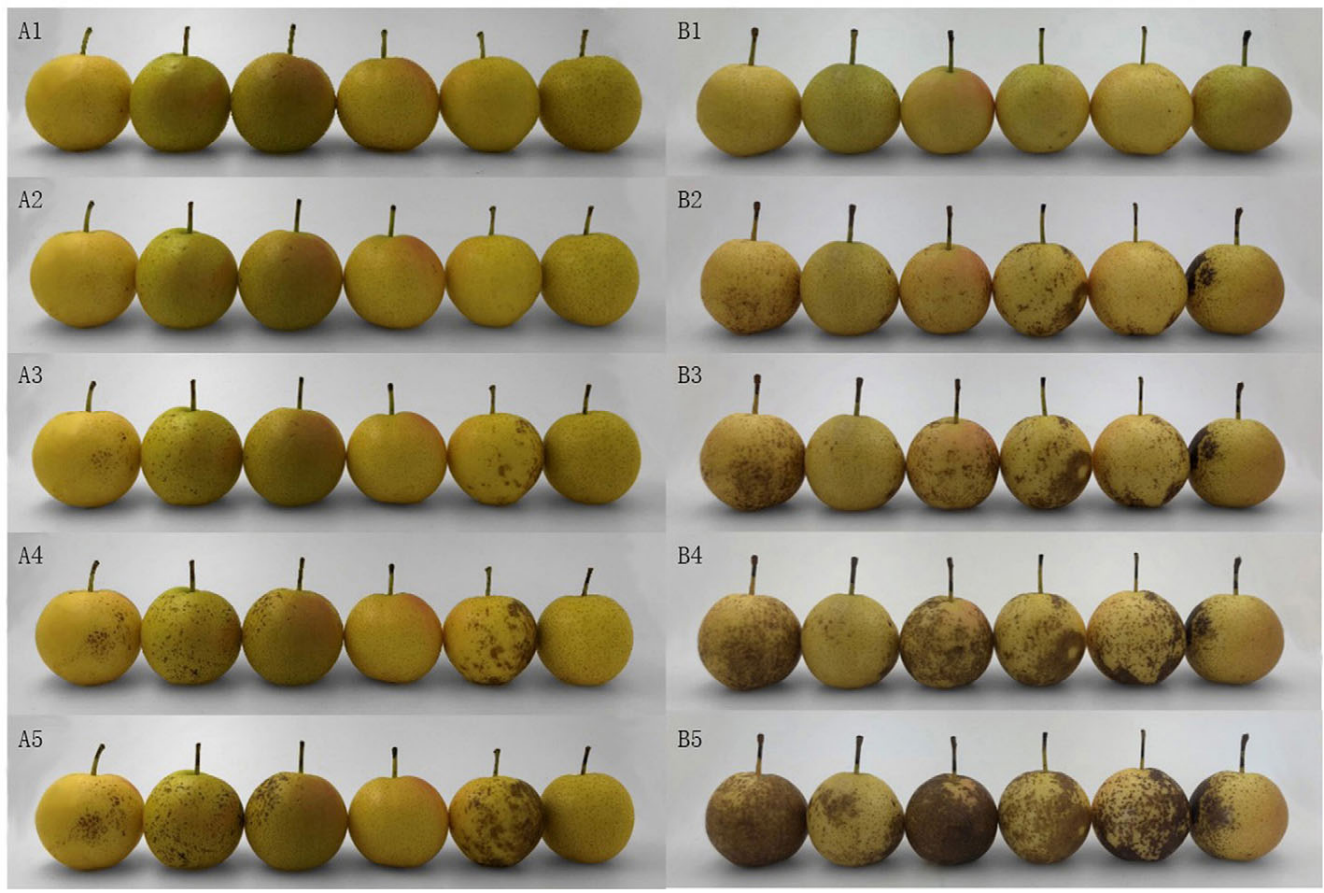
Pericarp browning during shelf life of CaCl_2_-treated (A1, A2, A3, A4, A5) and control (B1, B2, B3, B4, B5) ‘Nanguo’ pears after 180 days of cold storage. 1, 2, 3, 4, and 5 represent 0, 3, 6, 9, and 12 days of shelf life, respectively.

**Figure 2:**
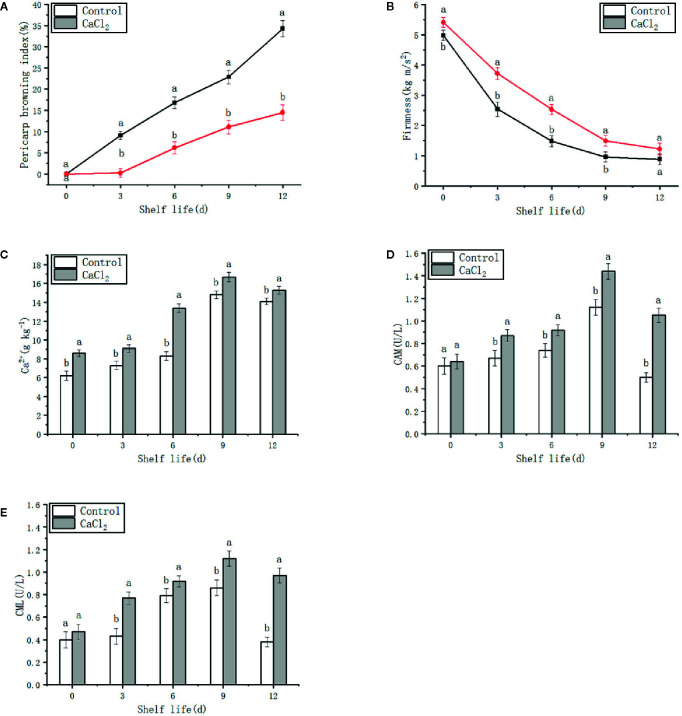
Pericarp browning index **(A)**, firmness **(B)**, Ca^2+^ **(C)**, CaM content **(D)** and CML content **(E)** in ‘Nanguo’ pear fruit during the 0, 3, 6, 9, and 12 days at 20°C after cold storage. Means ± SE of three replicate experiments are shown. Means with different letters are significantly different between the treatments and control (*p*< 0.05).

Pericarp browning of the stored fruits was visible in the untreated pears by day 3 on the shelf, and injury increased rapidly thereafter (**Figure 1**). In contrast, pears treated with CaCl_2_ began to show slight browning on day 6 on the shelf, and the symptoms developed slowly (**Figure 1**). The BI showed a similar trend, and was lower for the treated fruits than for the controls over the shelf period (**Figure 2A**). Thus, CaCl_2_ treatment retarded the occurrence and development of pericarp browning.

### Firmness Changes

Fruit softened gradually during the shelf period. CaCl_2_ treatment had little effect on fruit firmness until day 180 of storage. In the first nine days after storage, the firmness of the treated fruits was higher than that of the controls, while the fruit ripeness tended to be similar on day 12 (**Figure 2B**).

### Ca^2+^ Content and Distribution

Ca^2+^ content in both treated and untreated samples showed a similar trend (**Figure 2C**), gradually increasing until day 9 and declining thereafter. However, Ca^2+^ content in treated fruits was higher than that in the controls from days 0 to 12.

**Figure 3:**
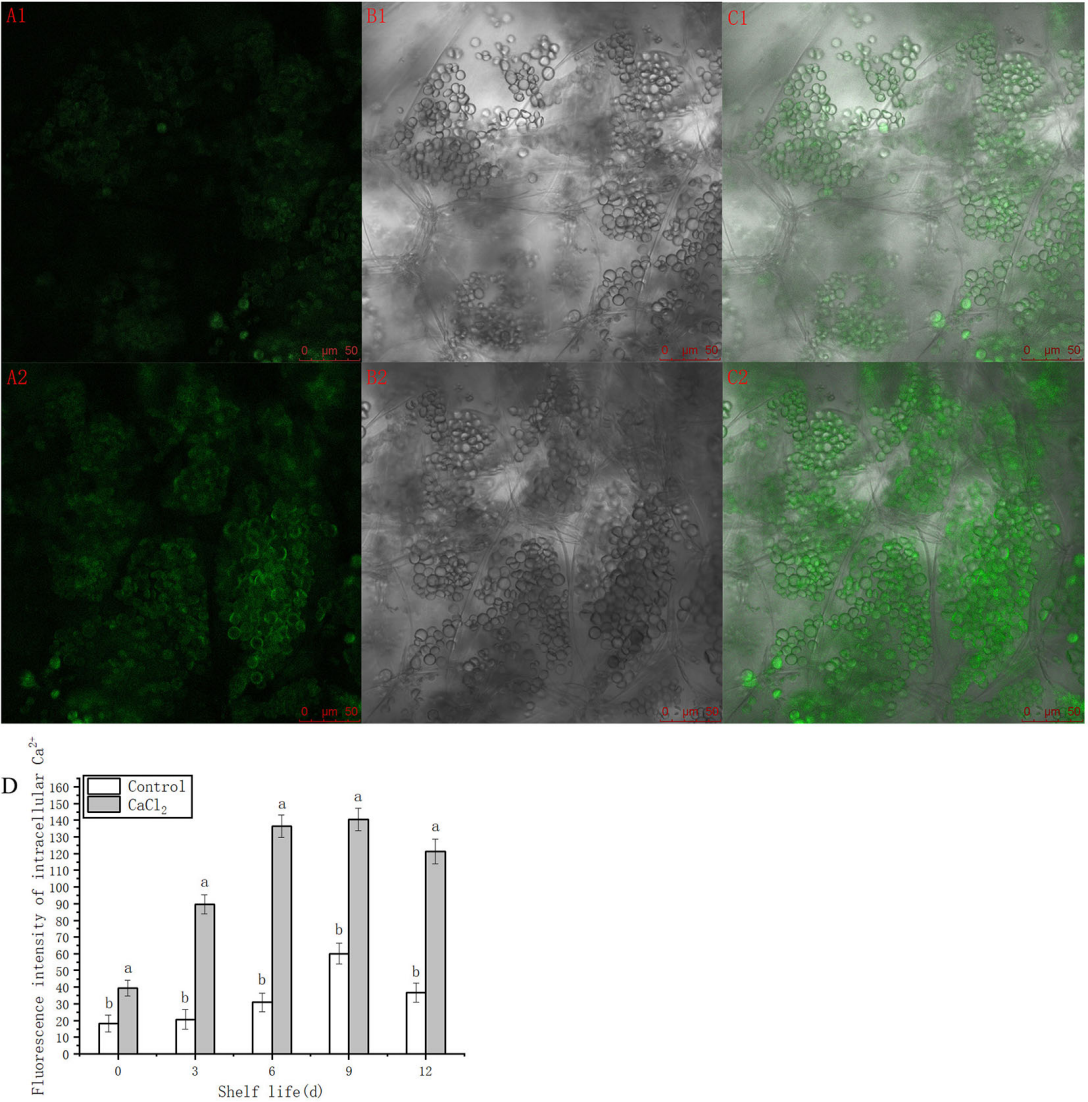
Dynamic change and cytochemical localization of Ca^2+^ in refrigerated but untreated (A1, B1, C1) and refrigerated with CaCl_2_-treated (A2, B2, C2) samples in the same magnification. **(A)** Ca^2+^ fluorescence signal characteristic excited by argon-ion laser sources. **(B)** ‘Nanguo’ pear pericarp structure scanned in transmitted light field. **(C)** Combination of **(A, B)**. **(D)** Semi quantification of the fluorescence intensity in ‘Nanguo’ pear pericarp during the 0, 3, 6, 9, and 12 days at 20°C after cold storage by ImageJ software. Standard deviation values are given. Values are means ± SE of three independent experiments, and five samples were assessed for each treatment. Means with different letters are significantly different between the treatments and control (*p* < 0.05).

To further investigate the distribution of Ca^2+^ in the cells, laser scanning confocal microscopy was used to observe intracellular Ca after 180 day of refrigeration, and the fluorescence intensity was analyzed using ImageJ software. During the shelf period, Ca^2+^ fluorescence intensity in the pericarp of the CaCl_2_-treated fruit was significantly higher than that in the untreated samples, which indicated that the treatment increased intracellular Ca^2+^ content. The pericarp cells of the CaCl_2_-treated samples appeared tight and clearly visible under the confocal microscope, with higher fluorescence intensity, and the fluorescence signal was concentrated in the tight parts of the cells (**Figure 3**). In addition, the semi-quantitative fluorescence intensity histogram showed that CaCl_2_ treatment significantly increased the Ca^2+^ content in the fruit peel throughout the shelf-life period (**Figure 3D**).

### CAM and CML Content

No noticeable differences in the CAM or CML contents were found between the two groups of the fruits on the day of removal from cold storage (**Figures 2D, E**). However, during the shelf period after the 180-day cold storage, both CAM and CML contents were significantly higher in the CaCl_2_-treated fruits than those in the control samples. The CAM and CML contents were higher later in the experimental shelf period, when they were more than twice those of the control samples. Therefore, CaCl_2_ treatment increased the CAM and CML contents.

### GABA Content

**Figure 4:**
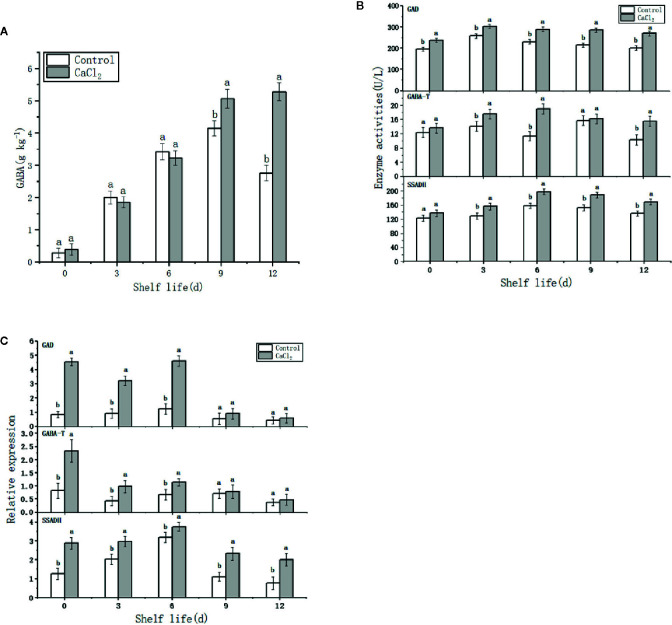
GABA **(A)**, GAD, GABA-T and SSADH activities **(B)** and relative expression of GAD, GABA-T, and SSADH **(C)** in “Nangou” pear fruit during the 0, 3, 6, 9, and 12 days at 20°C after cold storage. Means ± SE of three replicate experiments are shown. Means with different letters are significantly different between the treatments and control (*p*<0.05).

GABA content was relatively low during the early shelf life period after storage, and there was no obvious difference between fruit of the two groups (**Figure 4A**). However, with time after storage, GABA content in CaCl_2_-treated fruit increased gradually, particularly over the period from day 6 to 9, remaining at a high level until the end of the shelf-life period. Conversely, GABA content in untreated fruit initially increased but then decreased. GABA content in the CaCl_2_-treated fruit was higher than the control samples by nearly 92% on day 12.

### Activity and Related Gene Expression of GAD, GABA-T, and SSADH

GAD activity in CaCl_2_-treated samples remained higher than that in the control fruits after storage (**Figure 4B**). On the day of removal from cold storage, there was no noticeable difference in the activities of GABA-T or SSADH between the treated and untreated fruits. Thereafter, the GABA-T and SSADH activities were higher in the treated samples than those in the controls, with the highest values observed on day 6, when they were 68% and 25% higher than those in the control group, respectively. Thus, CaCl_2_ treatment promoted the synthesis of GABA as compared to the untreated fruits.

The expression of GABA synthesis-related genes reflects the potential capacity of GABA synthesis. The relative expression levels of the *GAD* and *GABA-T* genes in control fruits remained at a low level over the entire shelf period, whereas very high expression levels were observed in CaCl_2_-treated fruits from the day of removal from storage until the ninth day on the shelf (**Figure 4C**). The trend of the *SSADH* gene expression was similar to that of the corresponding enzyme activity; thus, it initially increased and then declined. Furthermore, the relative expression level of the *SSADH* gene in the CaCl_2_-treated samples was higher than that in the control fruits throughout the shelf period.

### Mitochondrial Ultrastructural Changes

**Figure 5:**
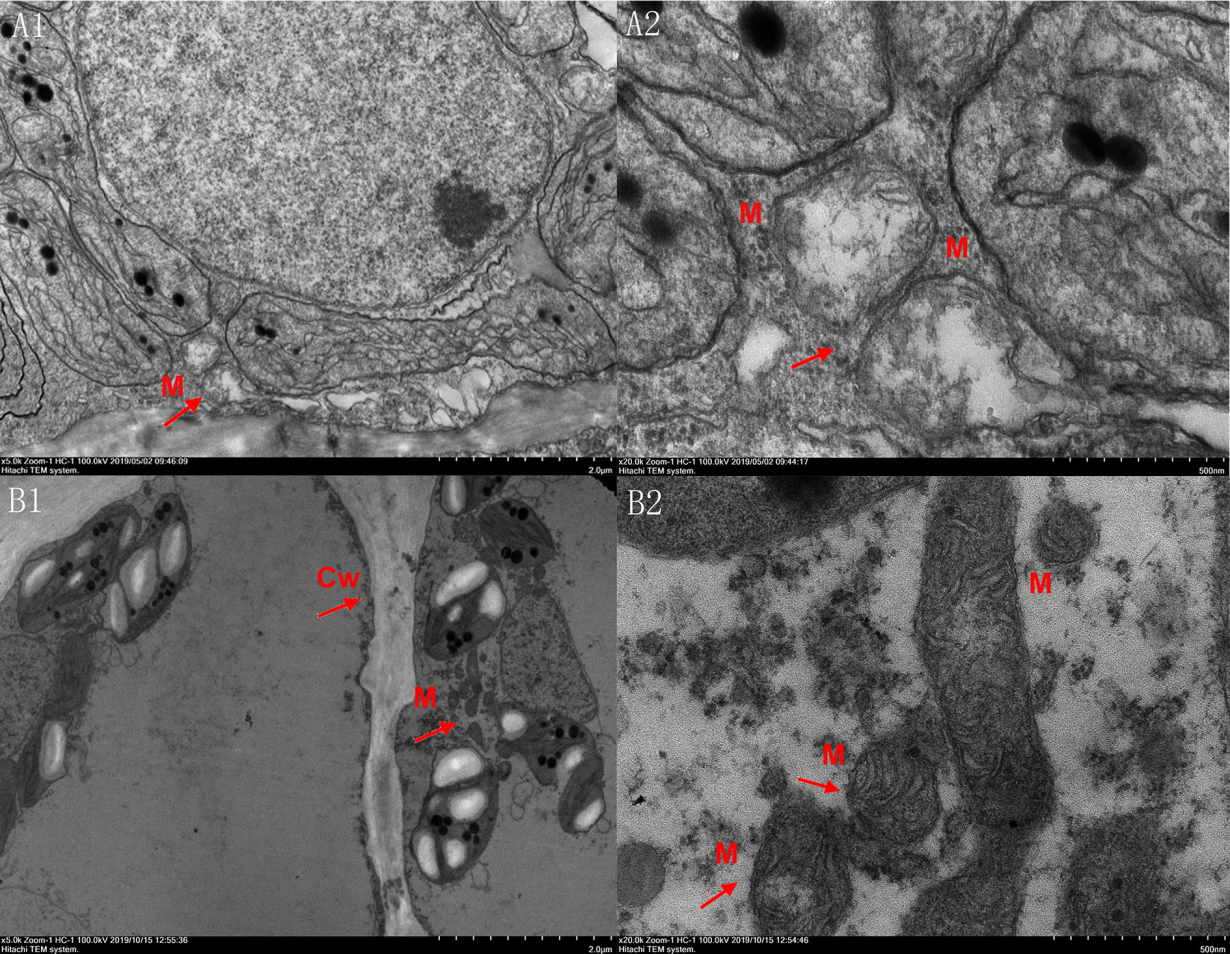
Ultrastructure in refrigerated but untreated (A1, A2) and refrigerated with CaCl_2_-treated (B1, B2) samples with the same maturity in different magnification. M, mitochondria; Cw, cell wall; Magnification power: A1, B1. ×5000; A2, B2. ×20,000.

Observation of the ultrastructure of mitochondria from the pericarp tissues of fruits on day 12 of the shelf period after storage, using TEM, revealed that the lumen of the mitochondria had shrunk. Mitochondrial inner ridges became blurred, disordered, and tended to fuse, and mitochondrial vacuolation occurred. Meanwhile, in the treated fruits, the mitochondrial ultrastructure appeared clear and intact, the matrix density was high, and the mitochondrial structure was clearly visible (**Figure 5**). Based on these results, it was concluded that CaCl_2_ treatment maintained the integrity of the mitochondrial structure in the pericarp of ‘Nanguo’ pears after long-term cold storage.

## Discussion

To explore the effect of calcium on the GABA branch, mitochondrial structure, and peel browning, peel browning was observed and the calcium content, mitochondrial microstructure, and the activities and the associated gene expression of a series of GABA-related anabolic enzymes were analyzed. Calcium plays a key role in the selective absorption of ions, signal transmission, and stress resistance in plants. At the cellular level, calcium has important regulatory functions in cell division, polarity formation, growth, differentiation, and apoptosis. It maintains cell wall stability, cell membrane structure, regulates the membrane-bound proteins, and participates in the regulation and control of many physiological and biochemical reactions of plant cells. Various stimuli, such as mechanical stimulation, low temperature, red light, plant hormones, fungal elicitors, hypoxia, and water stress have been shown to affect plant tissues, and the initial reaction almost always causes changes in the intracellular Ca^2+^ concentration (Bush, 1995). Ca^2+^ has been hypothesized to play an important role in plant resistance to stress (Luan et al., 2002). Previous studies have shown that the concentration of Ca^2+^ increases under low temperature stress in both chilling-resistant *Arabidopsis* spp. and chilling-sensitive tobacco (*Nicotiana plumbaginifolia*), which presumably triggers the Ca^2+^ signals that precede defense responses to stress (Knight et al., 1996). Further, Ca^2+^ has been shown to positively influence membrane integrity and cell membrane permeability (Picchioni et al., 1996; Picchioni et al., 1998). Ferguson et al. (1985) reported increased absorption of Ca^2+^ by the mitochondria of papaya fruit under low temperature stress. Damage to the cytoplasmic membrane is thought to be one of the most important causes of browning of fruit under cold storage, because damage to the cell membrane can lead to a series of secondary reactions, including the obstruction of energy production in the cytoplasm and the accumulation of toxic compounds (Rui et al., 2010). In the present study, the contents of Ca^2+^ and calmodulin in the pears were increased by Ca^2+^ treatment, which significantly increased the GABA content and effectively promoted the formation and metabolism of the GABA branch.

As a signaling molecule, GABA is widely distributed in prokaryotes and eukaryotes (Breitkreuz et al., 1999), playing an important role in resisting biotic and abiotic stress and participating in osmotic regulation in plants (Kumar et al., 2019). Our previous studies have shown that exogenous GABA treatment increases the endogenous GABA content of refrigerated ‘Nanguo’ pears, reducing ROS content and maintaining enzyme activity and the operation of the mitochondrial oxidative-defense system (Li J. X. et al., 2019). The GABA shunt contains three enzymes, the first of which is GAD, which initiates the decarboxylation of glutamate in the cytosol (Fait et al., 2005). GABA synthesis is terminated in the mitochondria by GABA decomposition into SSA and its conversion to succinic acid, which enters the TCA cycle and produces NADH (Fait et al., 2005; Wang et al., 2019). GABA is converted to succinic acid by the GABA shunt pathway to supplement the disrupted TCA cycle under stress conditions. SSADH is a key enzyme in this metabolic pathway (Bouché and Fromm, 2004). GAD activity is regulated by Ca^2+^ and CML. Many studies have shown that GABA levels increase rapidly in plants to cope with various forms of stress. Further, Bai et al. (2008) and Su et al. (2007) found that promoting GAD activity helps stimulate GABA accumulation. In the present study, Ca^2+^ treatment before storage increased GABA synthesis enzyme-related activities and their relative expression levels, as well as endogenous GABA, Ca^2+^, and CML contents in ‘Nanguo’ pears removed from cold storage and during the latter part of the experimental shelf period. The GABA shunt thus was prompted to assist in the normal TCA cycle operation in resistance to cold stress.

Mitochondria perform oxidative metabolism in eukaryotes and are the site of the ultimate oxidation of sugars, fats, and amino acids for release of energy. The mitochondrial matrix contains all the enzymes required for the TCA cycle, and the common pathway for final substrate oxidation in the mitochondria is the TCA cycle, followed by oxidative phosphorylation. Therefore, mitochondrial structure and membrane integrity play a vital role in maintaining normal plant metabolism to cope with environmental stress. In the current study, the integrity of mitochondria in untreated fruits was disrupted after 180 days of cold storage. GABA treatment has been shown to effectively maintain mitochondrial membrane permeability and structural integrity (Li Z. et al., 2019). From the perspective of GABA biosynthesis, the GABA synthesis-related key enzyme, GAD, was regulated by exogenous Ca^2+^ treatment. After Ca^2+^-treated fruit was placed in cold storage for 180 days, the mitochondrial structure in cells of the peel tissue was clear and complete. Consequently, the integrity of mitochondrial structure was maintained, and browning of ‘Nanguo’ pears was effectively alleviated.

## Conclusion

**Figure 6:**
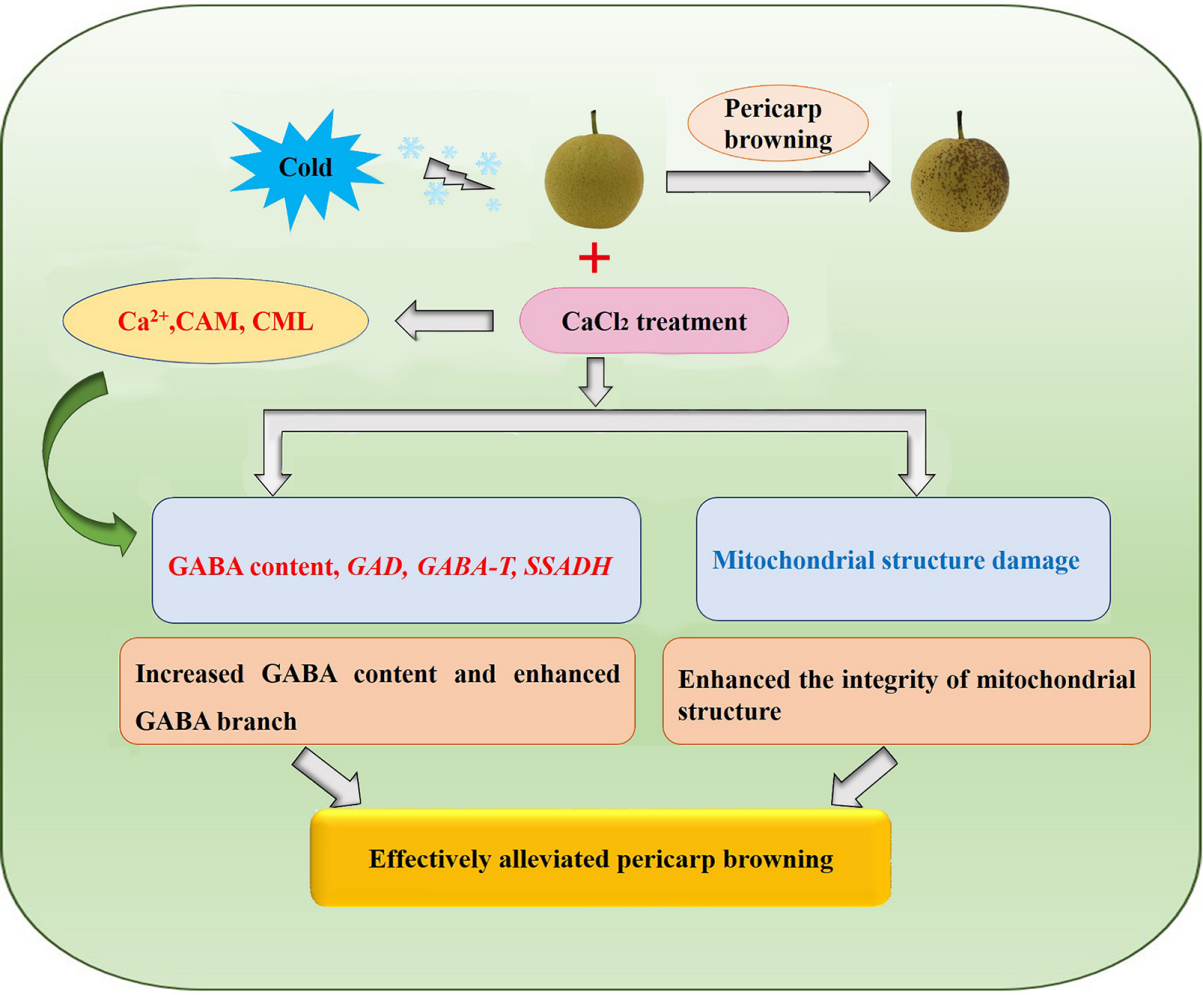
Proposed regulation module of pericarp browning alleviation through CACl_2_ treatment. CaCl_2_ treatment increased the content of Ca^2+^ and calmodium content in ‘Nanguo’ pear, thereby promoting GABA synthesis, increasing the activity and relative expression of *GAD, GABA-T* and *SSADH in* GABA shunt anabolism, and reducing the damage of mitochondrial structure caused by long-term cold storage, and finally ameliorating the pericarp browning. Red words represent promotion, whereas the blue ones represent inhibition. Details are described in the text.

Our results showed that exogenous Ca^2+^ delayed browning of the pericarp of ‘Nanguo’ pears upon removal from cold storage. The statistical analysis showed that exogenous CaCl_2_ treatment increased Ca^2+^ and CML content in the fruit, while increasing GAD activity, resulting in an increase in GABA content. This increased the activity of GABA-T and SSADH, and accelerated GABA biosynthesis according to external stress. In addition, TEM revealed that the mitochondrial structure in the pericarp of fruits treated with CaCl_2_ was clearer and more intact than that in the pericarp of the control fruits. Therefore, we concluded that CaCl_2_ treatment improved the ability of pear fruit to resist cold stress by regulating the GABA shunt and reduced browning of the pericarp of ‘Nanguo’ pears after long-term cold storage (**Figure 6**).

## Data Availability Statement

The raw data supporting the conclusions of this article will be made available by the authors, without undue reservation.

## Author Contributions

JL and SJ participated in the editing of the manuscript for this paper and the arrangement of the experimental process. BW and XZ provide help for the data analysis of the article. YZ and QZ supported the experimental operation. All authors of the study confirmed the accuracy of the content of the manuscript and the validity of the data.

## Funding

This work was supported by the National Natural Science Foundation of China (31971698).

## Conflict of Interest

The authors declare that the research was conducted in the absence of any commercial or financial relationships that could be construed as a potential conflict of interest.

## Abbreviations

GABA, γ-aminobutyrate; GAD, glutamate decarboxylase; TCA, tricarboxylic acid cycle; CAM, calmodulin; CML, calmodulin like protein; GABA-T, GABA transaminase; SSADH, succinate semialdehyde dehydrogenase.
