# Supplementary material for: Calcium Treatment Alleviates Pericarp Browning of ‘Nanguo’ Pears by Regulating the GABA Shunt After Cold Storage
Source: Front Plant Sci. 2020 Sep 15;11:580986. doi: 10.3389/fpls.2020.580986 (PMC7522215; doi:10.3389/fpls.2020.580986)
Supplement: Supplementary file 1 [file Table_1.docx]

**1.** The specific primers used in these experiments were designed using the Primer 6.0 software and the primer sequences for real-time q-PCR analysis are listed in Table 1

**Table 1**

Primers for real-time q-PCR Analysis

| Gene | Forward primer (5'-3') | Reverse primer (5'-3') | Product size (bp) |
| --- | --- | --- | --- |
| *PuGAD* (LOC103948174) | AGAGACAATGATAGAGGAAGG | CGTGAAACTTTCCGTCTTC | 149 |
| *PuGABA-T* (LOC103953698) | TGAGACAGAGGAGGAGTTC | GCTGGAGGAGGTATGACA | 134 |
| *PuSSADH* (LOC103964944) | CCAGAGACACCAATCACTATA | TCTTCATTCCAGCGTTCAA | 197 |
| *PuActin* (LOC103962665) | TTGGGATGGGTCAGAAGG | CTGTGAGCAGAACTGGGTG | 186 |

**2.** In our preliminary experiment, we treated the fruits with 1%, 2%, 4%, 8%, 16%, and 20% of CaCl_2_ during the pre-experiment, and found that the effect of 4% of CaCl_2_ on browning of refrigerated ‘Nanguo’ pear was the most obvious. The figure below is the pericarp browning index in stored pears under different concentrations of CaCl_2_ during shelf life.
